# Supplementary figures and images for: Mining integrated semantic networks for drug repositioning opportunities
Source: PeerJ. 2016 Jan 19;4:e1558. doi: 10.7717/peerj.1558 (PMC4736989; doi:10.7717/peerj.1558)

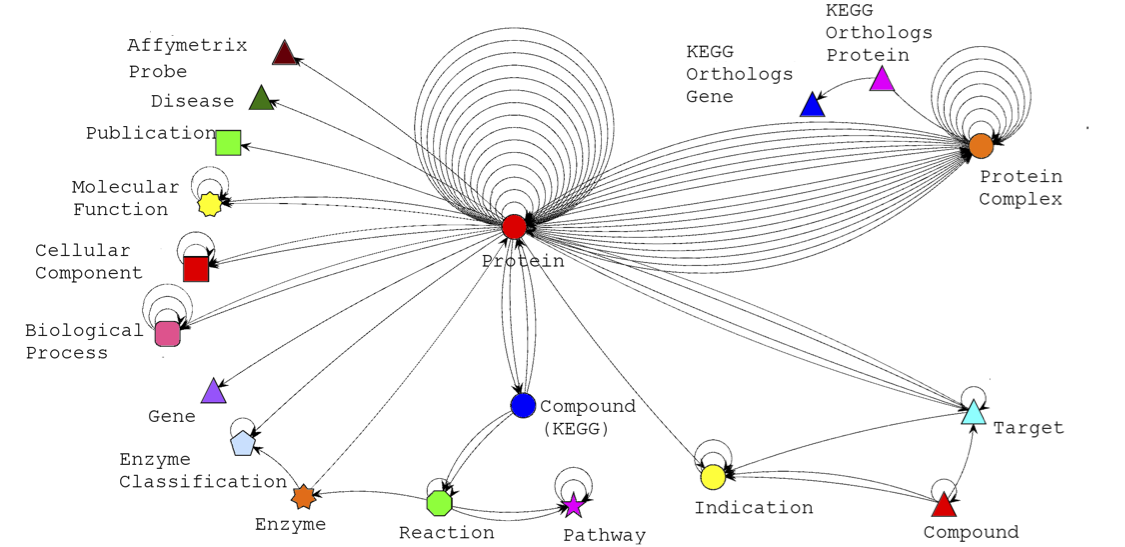

Supplement: Supplemental Information 1 — Notes: *Indicates data that was included in the updated dataset, used during this work. [file peerj-04-1558-s001.png]

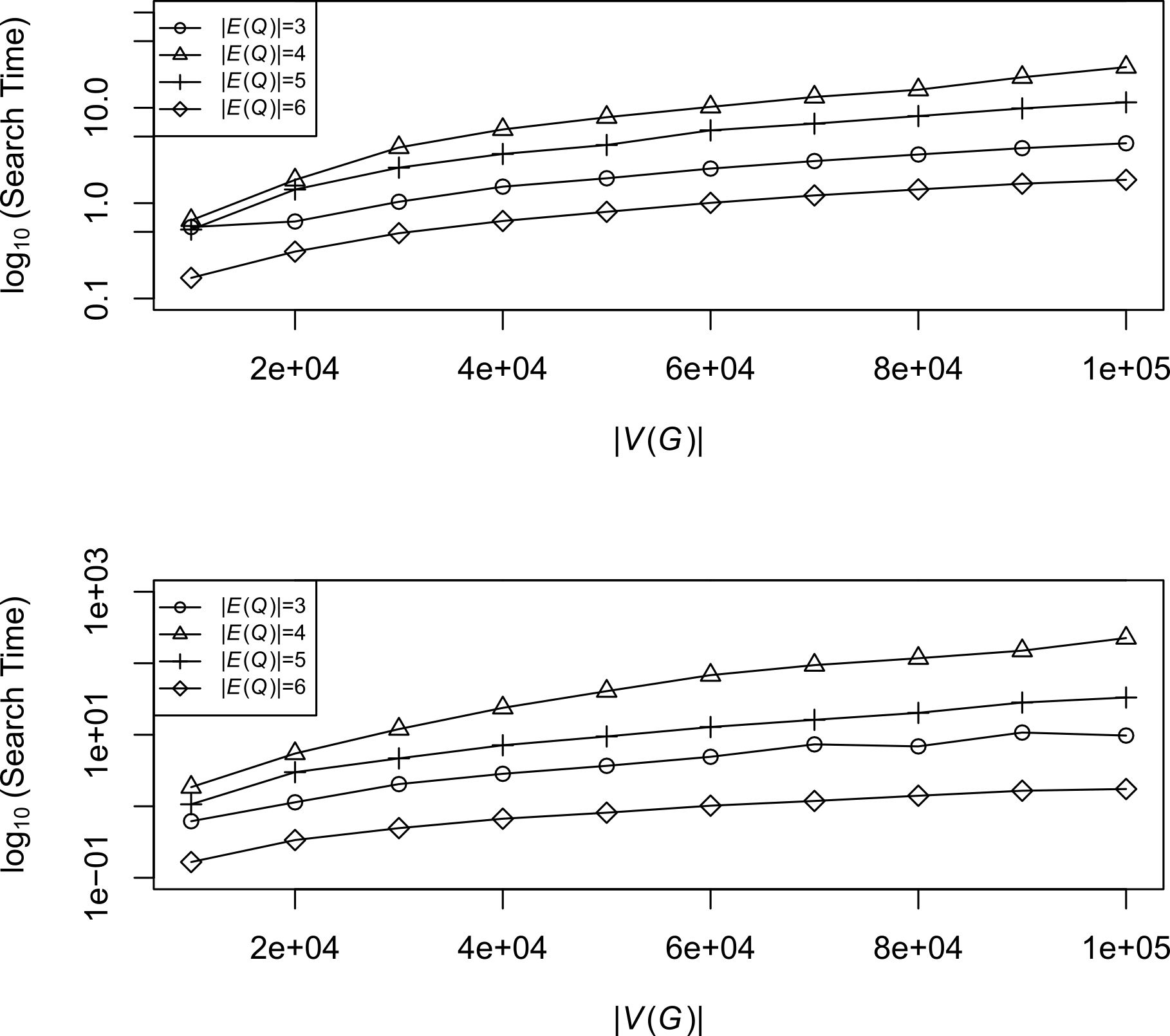

Supplement: Supplemental Information 2 — Note: Random semantic subgraphs were created with |V (Q)| of 4. Edgesets (|E (Q)|) of the subgraphs ranged from 3–6. Random target graphs were created with node sets ranging from 1 × 10^4^ to 1 x 10^5^. The algorithm used one of two parameters i) all elements of the match must be greater than ST (top) or ii) all elements must cumulatively be greater than the ST (bottom). [file peerj-04-1558-s002.png]

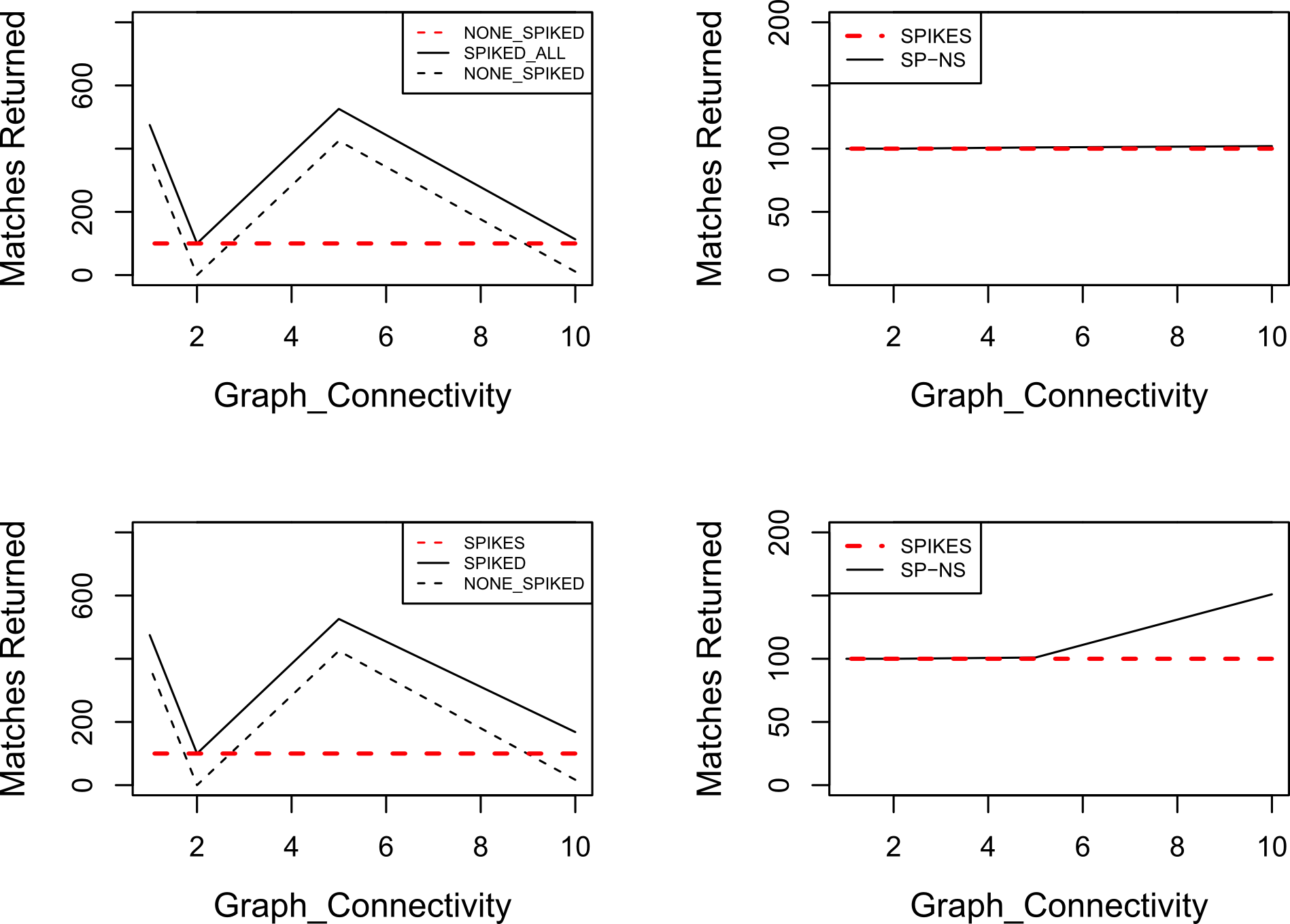

Supplement: Supplemental Information 3 — Note: Semantic subgraphs were created at random with a |V (Q)| of between 3 and 6. Runs were duplicated at least five times for each point in the graph, using the algorithm with two alternate parameters: i) all elements of the match must be greater than ST (top left and top right); and ii) all elements must cumulatively be greater than the ST (bottom left and bottom right). Graphs on the left show matches returned before and after spiking the target graph with 100 instances of the semantic subgraph that is to be searched for. Graphs on the right show the difference between the spiked (red) and non-spiked searches (black). [file peerj-04-1558-s003.png]

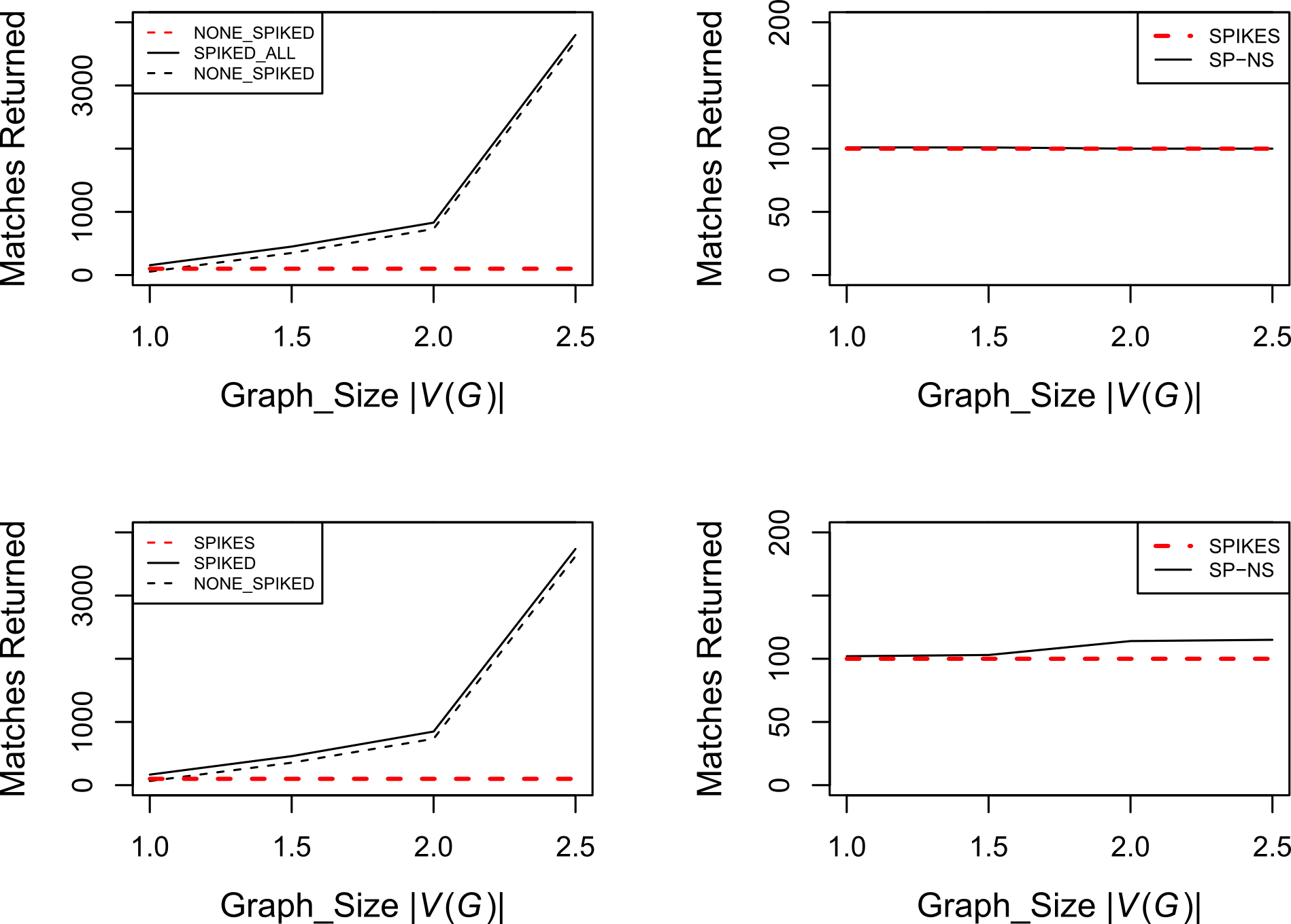

Supplement: Supplemental Information 4 — Note: Semantic subgraphs were created at random with a |V (Q)| of between 3 and 6. Runs were duplicated at least five times for each point in the graph, using the algorithm with two alternate parameters: i) all elements of the match must be greater than ST (top left and top right); and ii) all elements must cumulatively be greater than the ST (bottom left and bottom right). Graphs on the left show matches returned before and after spiking the target graph with 100 instances of the semantic subgraph that is to be searched for. Graphs on the right show the difference between the spiked (red) and non-spiked searches (black). [file peerj-04-1558-s004.png]

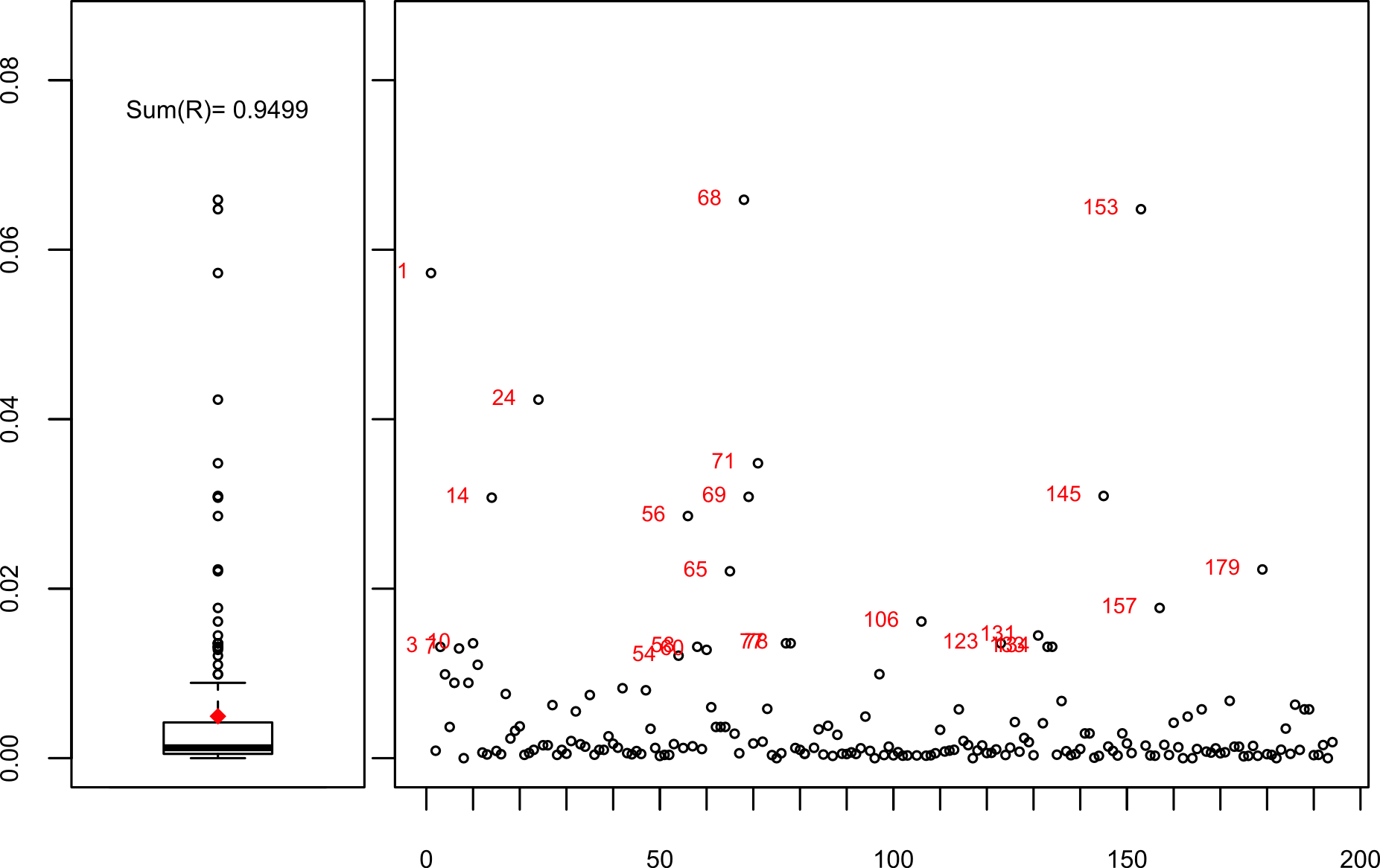

Supplement: Supplemental Information 5 — Left hand graph is a boxplot showing the semantic subgraph scores, with the mean score shown by a red point. Graph on the right shows the scores for subgraphs ranked based on ID. All subgraphs that scored > maximum are labelled with their ID. [file peerj-04-1558-s005.png]
