# Supplementary material for: Mining integrated semantic networks for drug repositioning opportunities
Source: PeerJ. 2016 Jan 19;4:e1558. doi: 10.7717/peerj.1558 (PMC4736989; doi:10.7717/peerj.1558)
Supplement: Supplemental Information 6 — Note: *Indicates data that was included in the updated dataset, used during this work. [file peerj-04-1558-s006.pdf]

ConceptClass	Nodes
Affymetrix_Probe	20,522
Biological_Process	19,046
Cellular_Component	2,731
Compound (DrugBank)	4,842
Compound (KEGG)	1,607
Disease	14,535
Enzyme_Classification	1,690
Enzyme	1,340
Gene	3,346
Kegg_Orthologs_Gene	2
Kegg_Orthologs_Protein	2
Molecular_Function	7,674
Pathway	436
Protein_Complex	196
Protein	22,665
Publication	45,059
Reaction	1,660
Target	3,500
Indication*	4,463
