# Supplementary material for: Mining integrated semantic networks for drug repositioning opportunities
Source: PeerJ. 2016 Jan 19;4:e1558. doi: 10.7717/peerj.1558 (PMC4736989; doi:10.7717/peerj.1558)
Supplement: Supplemental Information 7 — Note: *Indicates data that was included in the updated dataset, used during this work. [file peerj-04-1558-s007.pdf]

RelationType	Relations	Details
part_of_catalyzing_class	5,144	-
expressed_by	673	-
ubiquitinated_by	421	-
activated_by	4,430	-
has_function	50,922	-
regulated_by	4,333	-
adjacent_to	326	-
indirect_effect	778	-
part_of	5,332	-
produced_by	1,816	-
derives_from	210	-
has_participant	62,146	-
has_not_function	113	-
	4,813	-
repressed_by	4	-
dephosphorylated_by	410	-
share_intermediate	4,979	-
dissociated_from	51	-
interacts_with	37,166	Annotated with G-Sesame semantic similarity measures (no cutoff) (Du et al., 2009)
located_in	50,382	-
phosphorylated_by	1,537	-
binds_to	10,742	-
is_involved_in	15,400	-
published_in	109,061	-
is_a	48,726	-
has_similar_sequence	299,416	BLAST (E-value cutoff 1e-4)
inhibited_by	1,770	-
is_not_located_in	338	-
is_encoded_by	3,347	-
state_change_from	260	-
binds_to_encoding_mrna	22,514	-
protein_family	23,060	-
sim	12,256	2D-Tanimoto co-efficient calculated using (similarity cutoff 0.85) (O'Boyle et al, 2011)
is_part_of	573	-
member_is_part_of	1,957	-
consumed_by	1,845	-
participates_not	109	-
has_parent*	6,533	-
has_child*	2,018	-
may_treat*	3,744	-
may_prevent*	343	-
disgenet_involved_in*	16,098	-
