# Supplementary material for: Mining integrated semantic networks for drug repositioning opportunities
Source: PeerJ. 2016 Jan 19;4:e1558. doi: 10.7717/peerj.1558 (PMC4736989; doi:10.7717/peerj.1558)
Supplement: Supplemental Information 8 [file peerj-04-1558-s008.pdf]

Relation Type	From ConceptClass	To ConceptClass
part_of_catalyzing_class	Enzyme	Enzyme Classification
	Protein	Enzyme Classification
expressed_by	Protein Complex	Protein Complex
	Protein	Protein
	Protein	Protein Complex
ubiquitinated_by	Protein Complex	Protein Complex
	Protein	Protein
	Protein	Protein Complex
activated_by	Protein	Compound (KEGG)
	Protein	Protein
	Protein Complex	Protein
	Protein	Protein Complex
has_function	Protein	Molecular Function
regulated_by	Biological Process	Biological Process
adjacent_to	Pathway	Pathway
indirect_effect	Protein	Protein
	Protein Complex	Protein
	Protein	Protein Complex
part_of	Cellular Component	Cellular Component
	Molecular Function	Molecular Function
	Biological Process	Biological Process
	Protein	Protein Complex
produced_by	Compound (KEGG)	Reaction
derives_from	Pathway	Pathway
has_participant	Protein	Biological Process
has_not_function	Protein	Molecular Function
catalyzed_by	Reaction	Enzyme
repressed_by	Protein	Protein
	Protein	Protein Complex
dephosphorylated_by	Protein	Protein
	Protein Complex	Protein
share_intermediate	Reaction	Pathway
	Reaction	Reaction
	Protein	Protein
	Protein Complex	Protein
	Protein	Protein Complex
dissociated_from	Protein	Protein
	Protein Complex	Protein
interacts_with	Compound (KEGG)	Protein
	Protein	Protein
located_in	Protein	Cellular Component
phosphorylated_by	Protein Complex	Protein Complex
	Protein	Protein
	Protein	Protein Complex
	Protein Complex	Protein
binds_to	Compound (KEGG)	Protein
	Protein Complex	Protein Complex
	Compound (DrugBank)	Target
	Protein	Protein
	Protein	Protein Complex
is_involved_in	Protein	Disease
published_in	Protein	Publication
is_a	Enzyme	Protein
	Cellular Component	Cellular Component
	Molecular Function	Molecular Function
	Biological Process	Biological Process
	Protein	Target
	Target	Protein
has_similar_sequence	Target	Target
	Target	Protein

	Protein	Target
	Protein	Protein
inhibited_by	Protein	Compound (KEGG)
	Protein Complex	Protein Complex
	Protein	Protein
	Protein Complex	Protein
	Protein	Protein Complex
is_not_located_in	Protein	Cellular Component
is_encoded_by	KEGG Orth Protein	KEGG Orth Gene
	Protein	Gene
state_change_from	Protein	Protein
binds_to_encoding_mrna	Protein	Affymetrix Probe
protein_family	Enzyme Classification	Enzyme Classification
	Protein	Protein
sim	Compound (DrugBank)	Compound (DrugBank)
is_part_of	KEGG Orth Protein	Protein Complex
member_is_part_of	Reaction	Pathway
consumed_by	Compound (KEGG)	Reaction
participates_not	Protein	Biological Process
has_parent	Indication	Indication
has_child	Indication	Indication
may_treat	Compound (DrugBank)	Indication
may_prevent	Compound (DrugBank)	Indication
disgnenet_involved_in	Protein	Indication
	Target	Indication
