# Supplementary material for: Mining integrated semantic networks for drug repositioning opportunities
Source: PeerJ. 2016 Jan 19;4:e1558. doi: 10.7717/peerj.1558 (PMC4736989; doi:10.7717/peerj.1558)
Supplement: Supplemental Information 9 — Note: # SP refers to the number of drug-target associations captured in DBv3 and not in our dataset whose shortest path is captured via this subgraph. # Interactions refers to the number of drug-target associations inferred via S and # Unique refers to non-redundant inferred drug-target associations. The number of the inferred interactions that are captured in DBv3 are shown in the # Valid column. S score is to five decimal places. As we were using a semantic distance of 0.8 subgraphs that maintain a common topology and a relatively similar semantics (e.g. 62, 63 and 64) can return the same set of mappings. [file peerj-04-1558-s009.pdf]

S ID	#SP	# Inter	#Unique	# Valid	S score
1	687	18,271	12,420	711	0.05725
2	343	4,900,313	967,922	852	0.00088
3	273	27,499	21,389	281	0.01314
4	252	133,474	77,154	763	0.00989
5	178	85,139	66,224	244	0.00368
6	130	9,244	2,813	25	0.00889
7	98	14,597	3,090	40	0.01294
8	90	0	0	0	nan
9	90	9,244	2,813	25	0.00889
10	70	95,538	32,090	435	0.01356
11	47	34,362	17,519	193	0.01102
12	31	7,332,074	979,047	654	0.00067
13	31	51,606,527	2,195,719	958	0.00044
14	29	1,368	1,106	34	0.03074
15	29	10,537,633	882,229	753	0.00085
16	25	6,593,235	744,238	347	0.00047
17	23	104,479	16,111	122	0.00757
18	22	218,807	108,615	252	0.00232
19	19	173,227	80,300	260	0.00324
20	18	227,578	120,063	451	0.00376
21	17	58,798,303	2,051,596	809	0.00039
22	16	515,062,474	1,820,152	1,116	0.00061
23	16	136,787,222	735,508	722	0.00098
24	14	914	780	33	0.04231
25	13	86,935	53,659	82	0.00153
26	13	2,485,055	491,177	749	0.00152
27	11	247,239	92,623	580	0.00626
28	9	78,711,675	2,326,566	905	0.00039
29	9	698,182	201,116	197	0.00098
30	8	59,302,224	1,728,906	909	0.00053
31	8	772,850	244,370	495	0.00203
32	7	122,676	57,977	320	0.00552
33	6	1,620,033	340,527	561	0.00165
34	6	945,368	292,728	398	0.00136
35	6	100,677	27,106	202	0.00745
36	6	730,173,577	2,773,926	1,133	0.00041
37	5	136,787,222	735,508	722	0.00098
38	5	136,787,222	735,508	722	0.00098
39	5	21,063	17,045	44	0.00258
40	5	40,458	26,989	46	0.00170
41	5	2,102,113	533,000	658	0.00123
42	5	163,133	37,912	313	0.00826
43	4	5,093,736	849,741	508	0.00060
44	4	56,092,379	2,033,998	923	0.00045
45	4	2,903,808	709,792	597	0.00084
46	4	77,407,275	2,108,202	1,050	0.00050
47	4	125,517	12,352	99	0.00801
48	4	8,573	6,059	21	0.00347
49	3	639,873	105,171	127	0.00121
50	3	68,977,674	1,553,978	403	0.00026
51	3	78,711,675	2,326,566	905	0.00039
52	3	78,711,675	2,326,566	905	0.00039
53	3	1,791,973	140,171	233	0.00166
54	3	1,422	1,075	13	0.01209
55	3	524,958	281,027	338	0.00120
56	3	2,018	910	26	0.02857
57	3	242,592	105,576	149	0.00141
58	3	48,198	20,158	265	0.01315
59	3	492,497	84,607	91	0.00108
60	3	942	704	9	0.01278

61	3	18,153	2,993	18	0.00601
62	3	85,139	66,224	244	0.00368
63	3	85,139	66,224	244	0.00368
64	2	85,139	66,224	244	0.00368
65	2	2,102	952	21	0.02206
66	2	896	692	2	0.00289
67	2	13,499,739	979,060	569	0.00058
68	2	411	258	17	0.06589
69	2	2,035	908	28	0.03084
70	2	797,113	88,366	153	0.00173
71	2	667	431	15	0.03480
72	2	5,924	5,653	11	0.00195
73	2	90,606	11,683	68	0.00582
74	2	134,289,111	2,057,152	750	0.00036
75	2	67,253	9,975	0	0.00000
76	2	62,486,613	1,519,032	887	0.00058
77	2	95,538	32,090	435	0.01356
78	2	95,538	32,090	435	0.01356
79	2	2,950,327	92,390	111	0.00120
80	2	136,787,222	735,508	722	0.00098
81	2	541,963,227	1,197,081	630	0.00053
82	2	0	0	0	0
83	2	544,005	307,228	376	0.00122
84	2	492,882	21,444	73	0.00340
85	2	15,044,470	406,032	176	0.00043
86	1	303,662	99,815	382	0.00383
87	1	1,615,630	97,040	26	0.00027
88	1	248,061	98,255	271	0.00276
89	1	42,538,303	1,854,804	966	0.00052
90	1	82,719,532	2,056,629	1,004	0.00049
91	1	1,055,435	212,513	146	0.00069
92	1	140,271,069	1,919,147	956	0.00050
93	1	608,922	71,107	83	0.00117
94	1	12,156	2,648	13	0.00491
95	1	2,799,366	707,398	610	0.00086
96	1	0	0	0	nan
97	1	70,587	13,723	136	0.00991
98	1	105,489,172	1,034,057	380	0.00037
99	1	24,453	13,213	18	0.00136
100	1	983,613,015	2,766,763	931	0.00034
101	1	609,879	153,126	109	0.00071
102	1	382,576	13,582	4	0.00029
103	1	521,583,462	3,239,407	1,045	0.00032
104	2	0	0	0	0
105	1	521,583,462	3,239,407	1,045	0.00032
106	1	691	372	6	0.01613
107	1	613,742,795	3,801,900	1,118	0.00029
108	1	86,909,730	2,035,135	688	0.00034
109	1	62,486,613	1,519,032	887	0.00058
110	1	2,143,326	202,302	677	0.00335
111	1	498,915	105,472	83	0.00079
112	1	116,473	25,798	23	0.00089
113	1	48,634	26,236	26	0.00099
114	1	19,119	9,022	52	0.00576
115	1	4,666	2,461	5	0.00203
116	1	1,084,037	259,122	399	0.00154
117	1	0	0	0	nan
118	1	11,883,047	205,815	187	0.00091
119	1	1,844,246	378,387	568	0.00150
120	1	228,914	72,817	44	0.00060
121	1	297,313,162	1,262,289	798	0.00063

122	1	19,431,403	700,865	724	0.00103
123	1	95,538	32,090	435	0.01356
124	1	161,414,536	1,319,357	488	0.00037
125	1	1,869,792	428,086	531	0.00124
126	1	223,832	64,960	277	0.00426
127	1	27,088,403	1,072,321	821	0.00077
128	1	186,837	22,666	54	0.00238
129	1	29,785	15,329	29	0.00189
130	1	-465,959,328	3,392,238	1,175	0.00035
131	1	15,428	5,802	84	0.01448
132	1	170,371	32,516	134	0.00412
133	1	48,198	20,158	265	0.01315
134	1	48,198	20,158	265	0.01315
135	1	109,682,410	868,250	356	0.00041
136	1	3,132	890	6	0.00674
137	1	78,099,285	650,406	539	0.00083
138	1	488,409,710	3,003,840	1,047	0.00035
139	1	14,165,625	648,821	321	0.00049
140	1	16,897,972	748,248	812	0.00109
141	1	939,976	126,647	370	0.00292
142	1	939,976	126,647	370	0.00292
143	1	149,855	17,368	1	0.00006
144	1	781,116,124	3,053,867	847	0.00028
145	1	1,976	905	28	0.03094
146	1	1,527,829	92,472	127	0.00137
147	1	842,518	347,820	298	0.00086
148	1	978,027,001	3,012,567	943	0.00031
149	1	7,565	6,832	20	0.00293
150	1	1,713,918	356,681	620	0.00174
151	1	45,017,377	1,277,031	773	0.00061
152	2	0	0	0	0
153	1	401	247	16	0.06478
154	1	254,843	54,574	81	0.00148
155	1	1,377,363,946	2,767,620	902	0.00033
156	1	630,271,267	3,901,609	1,127	0.00029
157	1	14,700	3,892	69	0.01773
158	1	25,499,894	442,653	696	0.00157
159	1	76,317,908	2,187,104	829	0.00038
160	1	202,490	23,694	99	0.00418
161	1	218,568	82,796	107	0.00129
162	1	0	0	0	nan
163	1	12,156	2,648	13	0.00491
164	1	22,730	3,936	0	0.00000
165	1	16,897,972	748,248	812	0.00109
166	1	19,119	9,022	52	0.00576
167	1	609,201	195,219	152	0.00078
168	1	19,558,609	1,123,893	739	0.00066
169	1	17,405	3,454	4	0.00116
170	1	62,486,613	1,519,032	887	0.00058
171	1	1,956,776	136,316	95	0.00070
172	1	56,148	8,425	57	0.00677
173	1	945,368	292,728	398	0.00136
174	1	945,368	292,728	398	0.00136
175	1	507,601	13,591	3	0.00022
176	1	781,116,124	3,053,867	847	0.00028
177	1	149,144	109,789	159	0.00145
178	1	805,111,240	3,932,763	1,109	0.00028
179	1	12,912	5,478	122	0.02227
180	1	33,304	8,462	4	0.00047
181	1	78,711,675	2,326,566	905	0.00039
182	1	22	15	0	0.00000

183	1	44,002,766	655,137	658	0.00100
184	1	8,879	4,293	15	0.00349
185	1	58,608,849	1,623,471	833	0.00051
186	1	123,475	19,018	120	0.00631
187	1	194,932	146,006	143	0.00098
188	1	19,119	9,022	52	0.00576
189	1	19,119	9,022	52	0.00576
190	1	230,915,713	670,620	240	0.00036
191	1	453,400,362	2,543,461	989	0.00039
192	1	4,894,842	482,734	748	0.00155
193	1	53	44	0	0.00000
194	1	29,785	15,329	29	0.00189
