# Supplementary material for: Mining integrated semantic networks for drug repositioning opportunities
Source: PeerJ. 2016 Jan 19;4:e1558. doi: 10.7717/peerj.1558 (PMC4736989; doi:10.7717/peerj.1558)
Supplement: Supplemental Information 10 — Top 100 ranked drug-target associations inferred by DReSMin. [file peerj-04-1558-s010.pdf]

Drug	Target	S score
DB00661	Q13936	0.49211
DB01388	O00555	0.44378
DB01388	Q00975	0.43097
DB00420	P35348	0.39091
DB01224	P34969	0.38779
DB00777	P34969	0.38774
DB00661	O00555	0.38436
DB00661	Q00975	0.38181
DB01388	Q5SQC4	0.37526
DB00850	P34969	0.37383
DB00679	P34969	0.36830
DB00420	P34969	0.36825
DB00571	P21728	0.36085
DB00909	Q00975	0.35478
DB01202	Q00975	0.35478
DB00679	P41595	0.35036
DB00363	P34969	0.34799
DB00571	P35348	0.34664
DB01244	Q13936	0.34610
DB01202	O00555	0.34605
DB00909	O00555	0.34388
DB01202	Q5SQC4	0.34194
DB00909	Q5SQC4	0.34194
DB00368	P13945	0.34109
DB04841	O00555	0.34072
DB00571	P35368	0.34054
DB00755	P10828	0.33776
DB00653	Q9Y5Y9	0.33724
DB00679	P35368	0.33276
DB01202	Q13936	0.33190
DB00909	Q13936	0.33190
DB00571	P25100	0.33012
DB00850	P11229	0.33008
DB01244	O43497	0.32984
DB00508	P35348	0.32835
DB00433	P34969	0.32740
DB00433	P25021	0.32694
DB00285	P35348	0.32631
DB01224	P35348	0.32552
DB00653	Q14524	0.32519
DB00285	P35368	0.32399
DB06262	P13945	0.32387
DB00368	P21728	0.32302
DB00953	P21728	0.32218
DB00315	P21728	0.32218
DB01151	P35348	0.32208
DB04841	Q5SQC4	0.32155
DB04841	Q00975	0.32148
DB00755	P37243	0.32027
DB00669	P21728	0.31890
DB00777	P30939	0.31874
DB00420	P08908	0.31819
DB00508	P28335	0.31765
DB00668	P28222	0.31760
DB00661	Q5SQC4	0.31751
DB00363	P35368	0.31703
DB00508	P35368	0.31664
DB01151	P35368	0.31506
DB00679	P28221	0.31386
DB00246	P35368	0.31356

DB00508	P25021	0.31336
DB00623	P34969	0.31176
DB00433	P28222	0.31153
DB00800	P34969	0.31139
DB00420	P28222	0.31114
DB00996	O43497	0.31043
DB00508	P34969	0.31028
DB00850	P41595	0.30982
DB00589	P34969	0.30872
DB00960	P35348	0.30847
DB01624	P34969	0.30836
DB00777	Q13639	0.30676
DB00850	P28222	0.30670
DB00850	P28221	0.30652
DB01142	P21728	0.30564
DB00734	P41595	0.30516
DB00477	P35348	0.30493
DB00381	O43497	0.30492
DB00777	P28222	0.30430
DB00850	P08908	0.30384
DB00850	P35368	0.30359
DB01388	Q9Y5Y9	0.30328
DB00875	P34969	0.30314
DB00246	P34969	0.30150
DB01142	P35348	0.30088
DB00850	P08172	0.30051
DB00104	P41145	0.29994
DB00285	P21728	0.29893
DB00270	O95180	0.29888
DB00508	P28222	0.29858
DB00420	P25100	0.29852
DB00459	P10828	0.29829
DB00622	O95180	0.29822
DB00334	P34969	0.29818
DB00831	P35368	0.29817
DB00523	P10828	0.29813
DB01244	O95180	0.29757
DB00502	P34969	0.29740
DB00622	O43497	0.29726
