# Supplementary material for: Mining integrated semantic networks for drug repositioning opportunities
Source: PeerJ. 2016 Jan 19;4:e1558. doi: 10.7717/peerj.1558 (PMC4736989; doi:10.7717/peerj.1558)
Supplement: Supplemental Information 11 — Article provides a more concise description of the graph split component of DReSMin. [file peerj-04-1558-s011.pdf]

Algorithm 1 Graph Splitting

Input: Semantic Subgraph, Q

if $|V(Q)| \leq 4$ **then**
  $D = \{Q\}$
else
  Q is converted to an undirected graph
  if cliqueCheck (Q) **then**
    cliqueSplit (Q)
  else
    $ON = v_{max}(Q); v_1; v_2; max = -1$
    for $i \in V(\forall j(j \in V \wedge i \neq j))$ **do**
      if shortestPath $(i, j) > max$ **then**
        $max =$ shortestPath $(i, j); v_1 = i; v_2 = j$
      end if
    end for
    $D_1 =$ all nodes in $\delta(v_1, ON)$
    $D_2 =$ all nodes in $\delta(v_2, ON)$
    for $v \in V$ **do**
      if $v \notin (V(D_1) \cup V(D_2))$ **then**
        allocate left over nodes
      end if
    end for
    $D = \{D_1, D_2\}$
    while $|(V)d| \in D > 4$ **do**
      return Graph Splitting(d)
    end while
  end if
end if

Output: D

Graph Split

In Graph Splitting the shortest path is calculated using the implementation of Dijkstras shortest path algorithm (Dijkstra, 1959) provided by the JGraphT library. If a semantic subgraph is found to be a clique then an ON is picked at random and D is made up of a random partitioning of Q, such that $|V(D_1)|$ and $|V(D_2)|$ are equal if n is odd or $|V(D_1)| = (|V(D_2)| + 1)$ if n is even (ON is in both graphs). If Q is not a clique then semantic subgraph membership of remaining nodes depends upon the edges in which they participate. Given semantic subgraphs D_1 and D_2, node subgraph membership is allocated using the following rules:

1. If a node shares an edge with only one of D_1 or D_2 it will be allocated to that subgraph.

2. If a node shares an edge with nodes from both D_1 and D_2 it will be allocated to the subgraph with which it shares the greatest number of edges.

3. If a node shares an equal number of edges with nodes in D_1 and D_2 it will be allocated to the subgraph containing the fewest nodes.

4. If a node shares an equal number of edges with nodes in D_1 and D_2 and D_1 and D_2 have an equal number of nodes it will be allocated D_1.

Edges ($e \in E(Q)$) are then allocated to D_1 if both nodes of e, v_i, v_j, are $v \in V(D_1)$ or to D_2 if both nodes of e, vi, vj, are $v \in V(D_2)$. When v_i, v_j are not present in the same graph then e is not included in the split graphs. Instead e will be checked for during the mapping stage of the algorithm. If either of the subsequent graphs still have a nodeset greater than 3 after an initial split, the algorithm may be called again and the resulting subgraphs are searched for and results mapped systematically.

In Fig. 1 (below), it is clear that searching using a purely topological approach (Normal_0.0) is infeasible for a subgraph with a $|V(Q)|$ of 7 when the target graph reaches a nodeset size of 2 x 10^3. Performance is improved when searching using a semantic threshold of 0.8 (Normal_0.8). However, search time is still costly when the target graph reaches a nodeset size of 3 x 10^4. When Graph Splitting is used prior to a semantic search with a semantic threshold of 0.8 (Split), a search for semantic subgraphs with a $|V(Q)|$ of 7 can be completed on a large target graph (1 x 10^5) in a reasonable, finite time.

These results show that Graph Splitting allows us to search for subgraphs that were previously intractable using an exhaustive exact approach; both topologically and, to a lesser extent, semantically.

The Venn Diagrams (Fig. 2 below) demonstrate that the same results are obtained (i.e. the same set of matches are returned) when using Graph Splitting or a non-split approach.

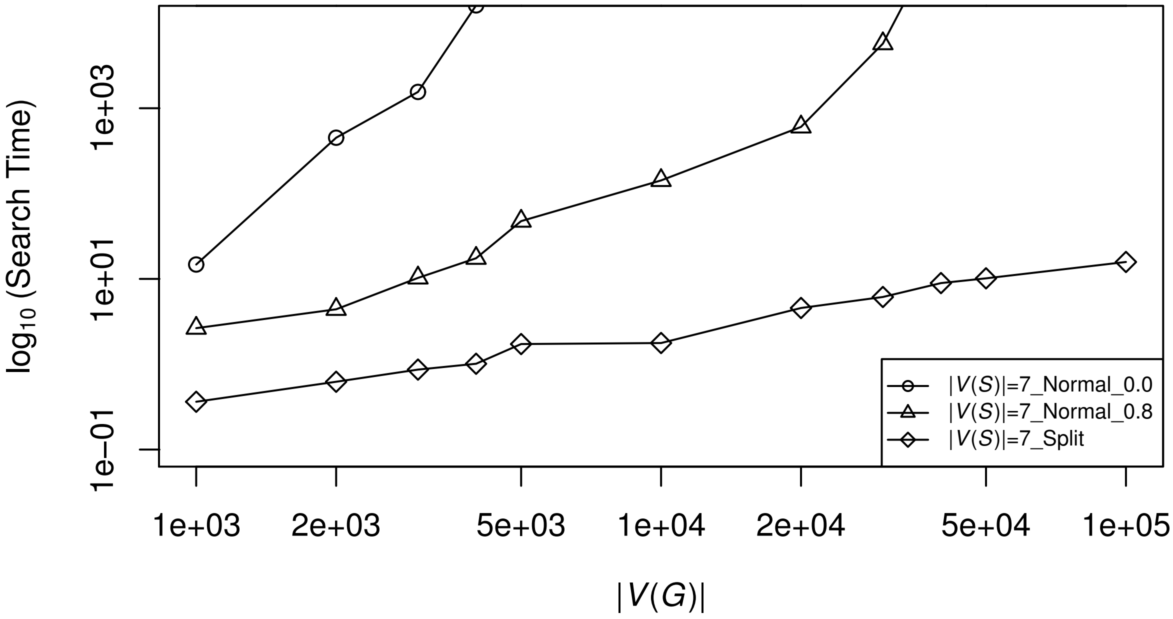

Figure 1: **Graph Splitting search time in comparison to a non-split approach.** *Note:* Semantic subgraphs were created at random with a $|V(Q)|$ of 7. A purely topological search was then completed without Graph Splitting (Normal_0.0). Semantic searches were then carried out without Graph Splitting (Normal_0.8), and finally, with Graph Splitting (Split). Searches were carried out on random target graphs where $|V(G)|$ was between 1 x10^3 and 1x 10^5.

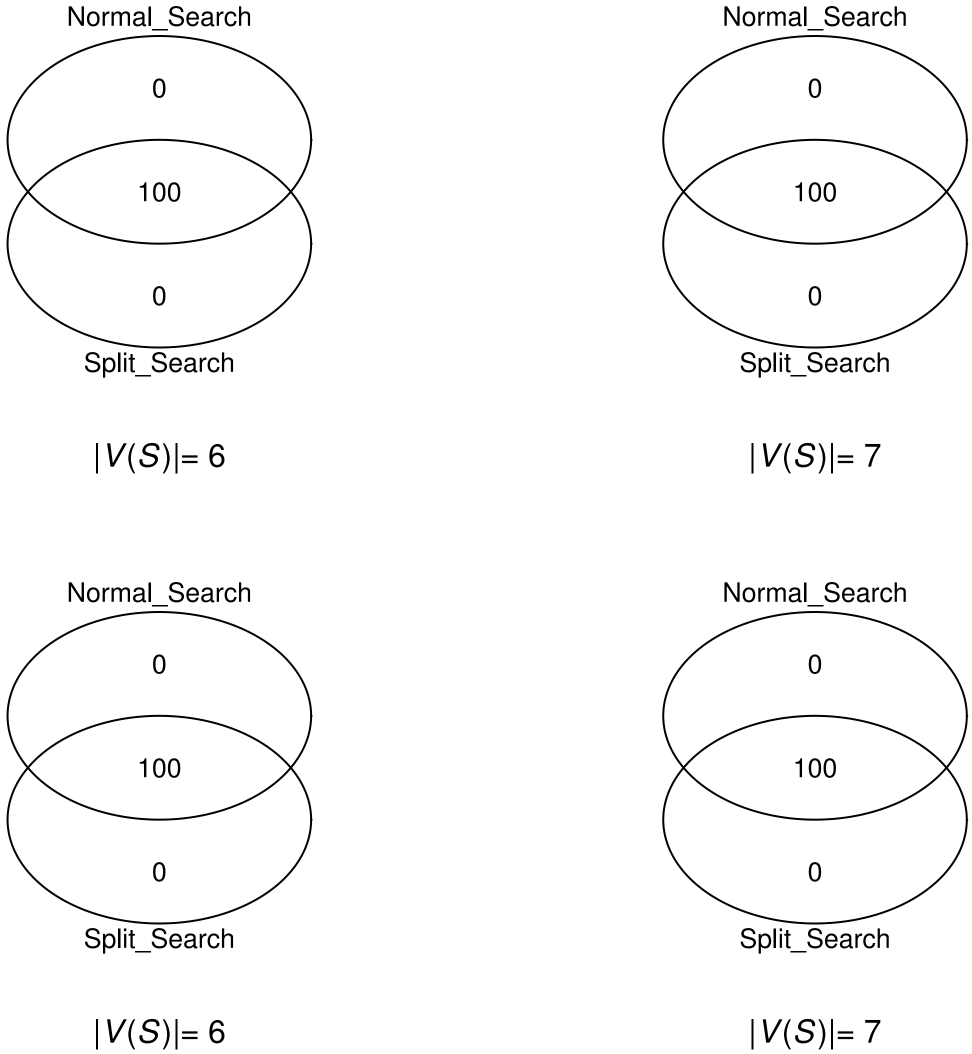

Figure 2: **Graph Splitting matches in comparison to matches returned using a non-split approach.** *Note:* Semantic subgraphs were created at random with a $|V(Q)|$ of 6 and 7, respectively. Semantic subgraphs were then searched for using either Graph Splitting (Split_search) or a non-split approach (Normal_Search) using the algorithm with one parameter; every element of the match needed to pass the ST (left). Returned sets of matches were compared and the percentage of matches between the two sets calculated. All tests were run using the algorithm with two alternate parameters i) where every element of the match needed to pass the ST (left), and ii) all elements had to cumulatively pass the ST (right).
