# Supplementary material for: Mining integrated semantic networks for drug repositioning opportunities
Source: PeerJ. 2016 Jan 19;4:e1558. doi: 10.7717/peerj.1558 (PMC4736989; doi:10.7717/peerj.1558)
Supplement: Supplemental Information 12 — Article provides an explanation of the semantic threshold (ST) employed during this work. [file peerj-04-1558-s012.pdf]

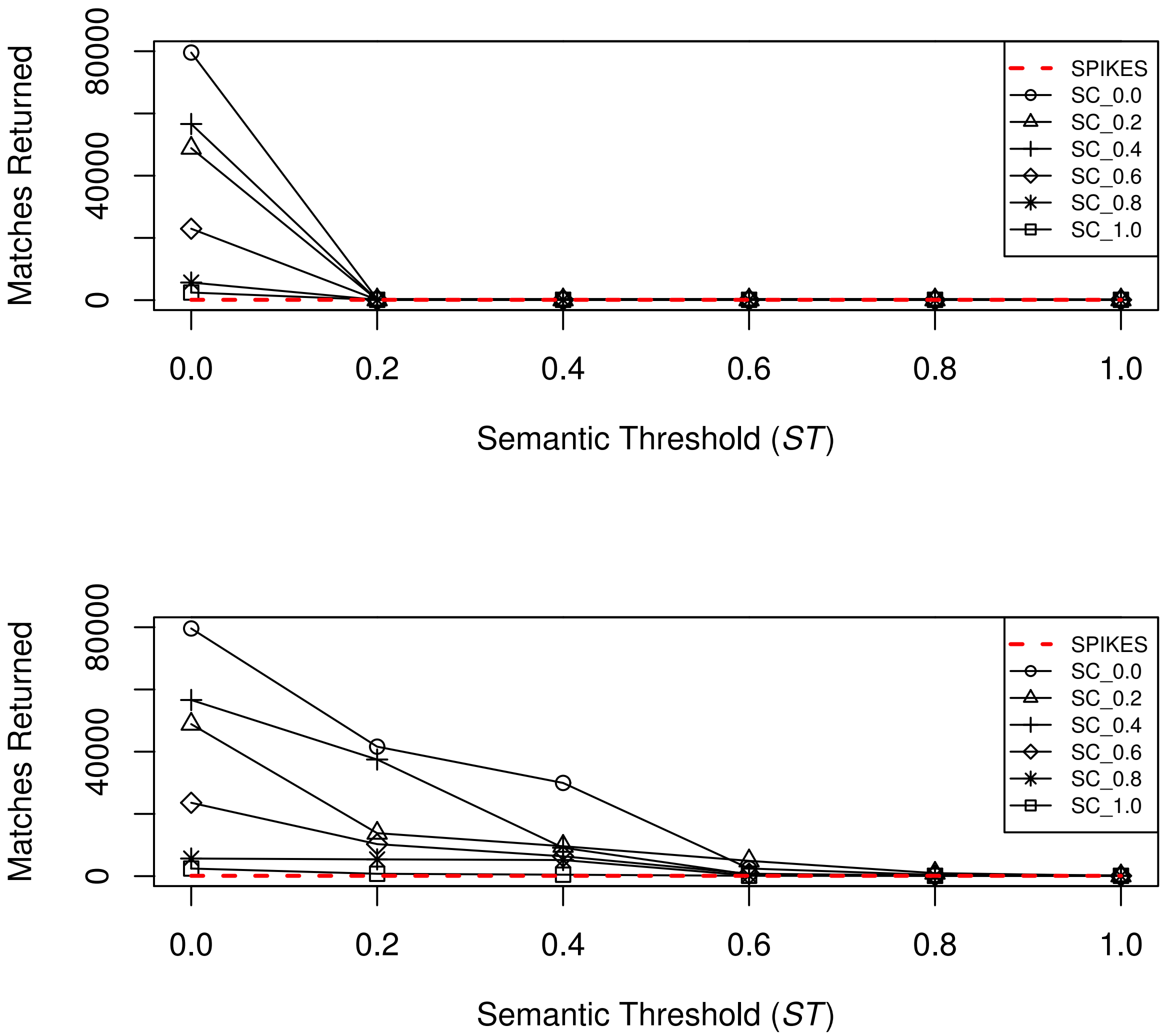

Figure 1: **Calculating semantic threshold (ST) to be used during searches.** *Note:* Creating semantic subgraphs with semantic complexities* ranging from 0-1, we see at which ST only the 100 spiked instances are returned. The algorithm was ran on random graphs with a node set size of 1 x 10^3 using two alternate parameters where i) every element of the match needed to pass the ST (left), and ii) all elements had to cumulatively pass the ST (right). When ST reaches 0.8 only very few matches that are not members of the spiked subgraphs are returned. Subgraphs were created at random with nodesets between 3 and 6.

*We define the semantic complexity (SC) of a semantic subgraph (Q) by the following:

$$SC(Q) = \frac{\sum_{v_i,v_j \in V(Q), v_i \neq v_j} SDC(v_i, v_j) + \sum_{e_i,e_j \in E(Q), e_i \neq e_j} SDC(e_i, e_j)}{|V(Q)| + |E(Q)|} \quad (1)$$
